# Focal treatment of prostate cancer using Focal One device: pilot study results

**DOI:** 10.1186/2050-5736-3-S1-O54

**Published:** 2015-06-30

**Authors:** Albert Gelet, Sebastien Crouzet, Olivier Rouviere, Flavie Bratan, Jean-Yves Chapelon

**Affiliations:** 1 Edouard Herriot Hospital, Lyon, France; 2 Hospices Civils de Lyon, Lyon, France; 3 Inserm, Lyon, France

## Background/introduction

Objectives: To evaluated the efficacy of Focal One device for focal treatment of localized prostate cancer.

## Methods

Material and method: Focal One^®^ is a device designed for the focal therapy of Prostate Cancer combing the necessary tools to visualize, target, treat and validate the focal treatment. MR volumes are imported through the hospital’s network. The operator defines the contours of the prostate and the regions of interests that have been confirmed as prostate tumors. The same contouring of the prostate is performed on the live ultrasound volume acquired by the transrectal probe. The software proceeds to an “elastic fusion”: the live Ultrasound volume is considered as the reference volume and the MR volume is smoothly deformed so the 3D contour of the prostate on the MR volume matches perfectly the contours of the prostate on the Ultrasound Volume. The same 3D elastic transformation is applied to the ROIs initially indicated on the MR image so they appear at the adequate position on the live Ultrasound Image, guiding the planning process. The Focal One is equipped with a new generation of HIFU probe able to electronically vary the focal point along the acoustic axis using a HIFU multi-element phase array transducer. During the HIFU energy delivery process, the operator sees a live ultrasound image of what is being treated and, if necessary, can readjust the treatment planning. At the end of the treatment process, a Contrast-enhanced Ultrasound volume is acquired showing the de-vascularized areas.

Teen patients with mono focal prostate cancer were treated between March 2013 and January 2014. HIFU treatment process was realized with the Focal One device using a 6 mm safety margin around the tumor. Contrast enhanced MRI is performed at day 2 after HIFU and Control biopsies guided with contrast-enhanced Ultrasound imaging were achieved one month after HIFU inside and in the rime of the treated area.

## Results and conclusions

Results: The mean age of patients was 65.8±5.5 years. The Clinical stage was T1 for 9 patients and T2a for 1 patient. The Gleason sum was 6 for 7 patients and 7(3+4) for 3 patients. The PSA value was 4.47± 3.7 ng/ml and the mean Prostate Volume was 50±23 cc. The mean treated volume was 14 cc (7.3-20.4) 28% of prostate gland. The mean nadir PSA value was 3.46±2 ng/ml. In all patients, targeted biopsies inside the treated area performed day 30 after the HIFU session demonstrated a complete destruction of the targeted tumor. No incontinence was observed. A partial loss of potency (IIEF <17) occurred in 2 patients.

Conclusions: Focal One device is able to achieve a complete destruction of small prostate cancer using an elastic magnetic resonance-ultrasound (MR-US) registration system for tumor location and HIFU treatment planning. Multicenter trial is in progress (30 patients).

**Figure 1:**
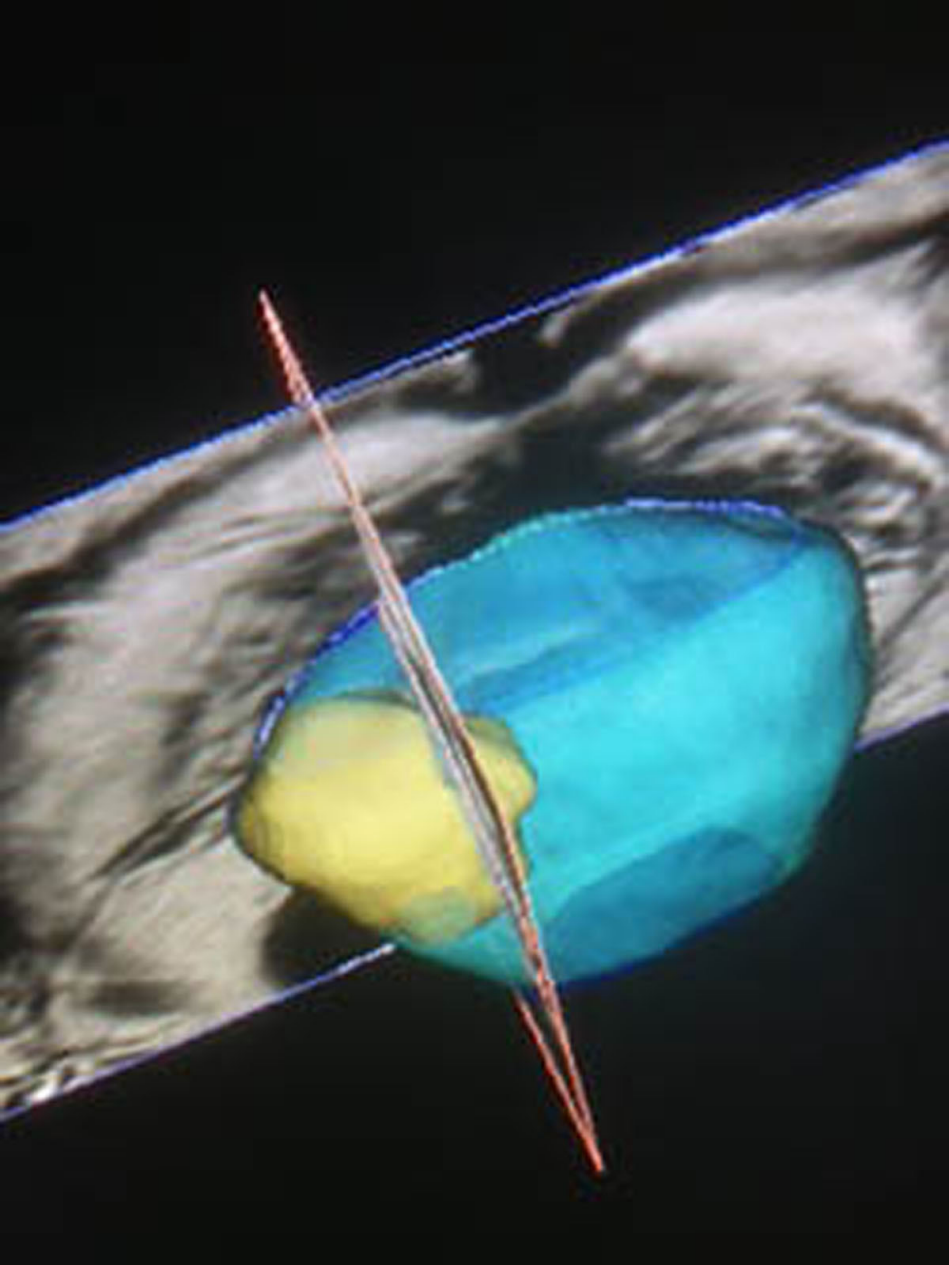
MRI registration with elastic fusion

**Figure 2:**
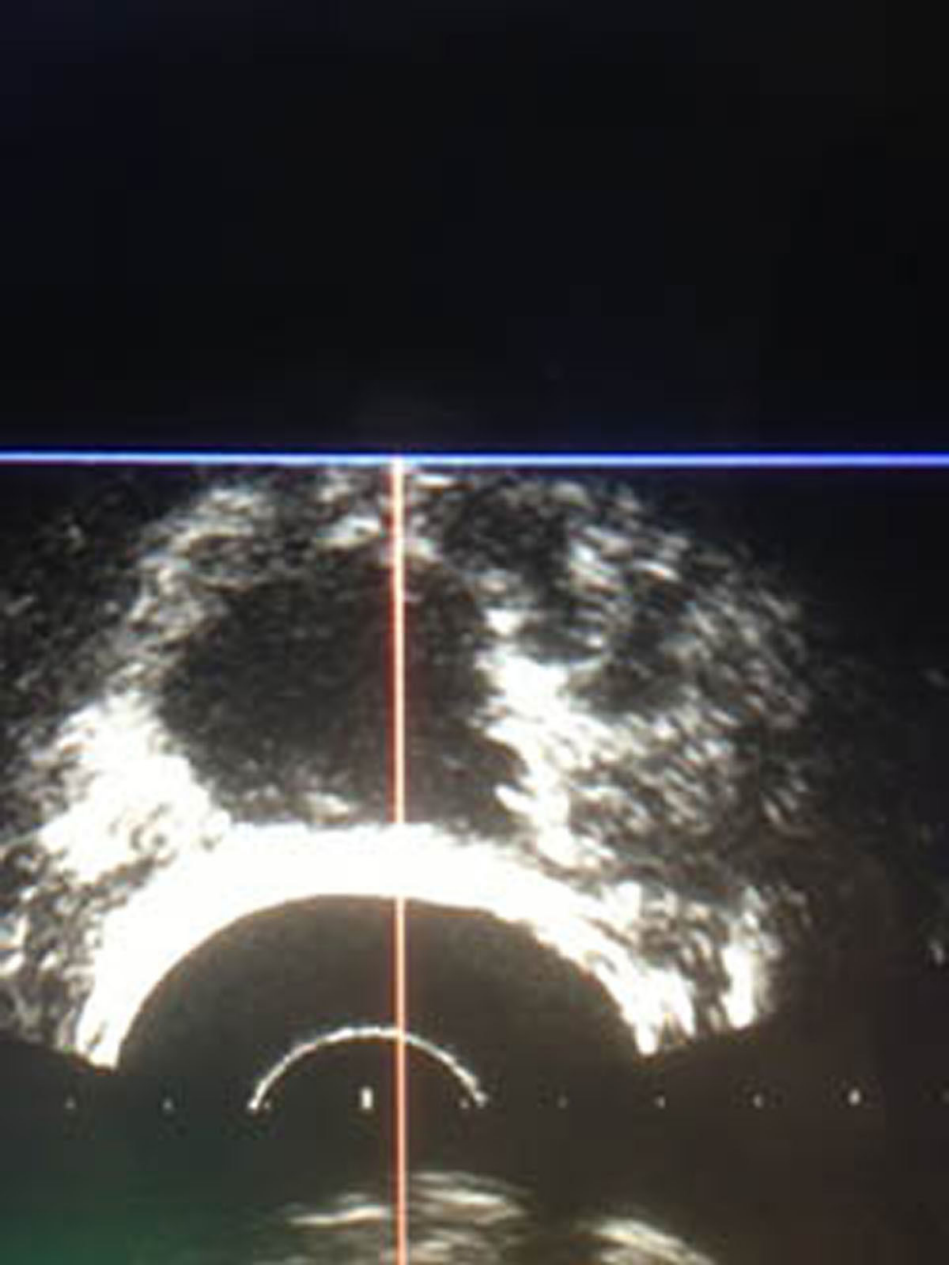
Contrast-enhanced Ultrasound showing the de-vascularized areas.

**Figure 3:**
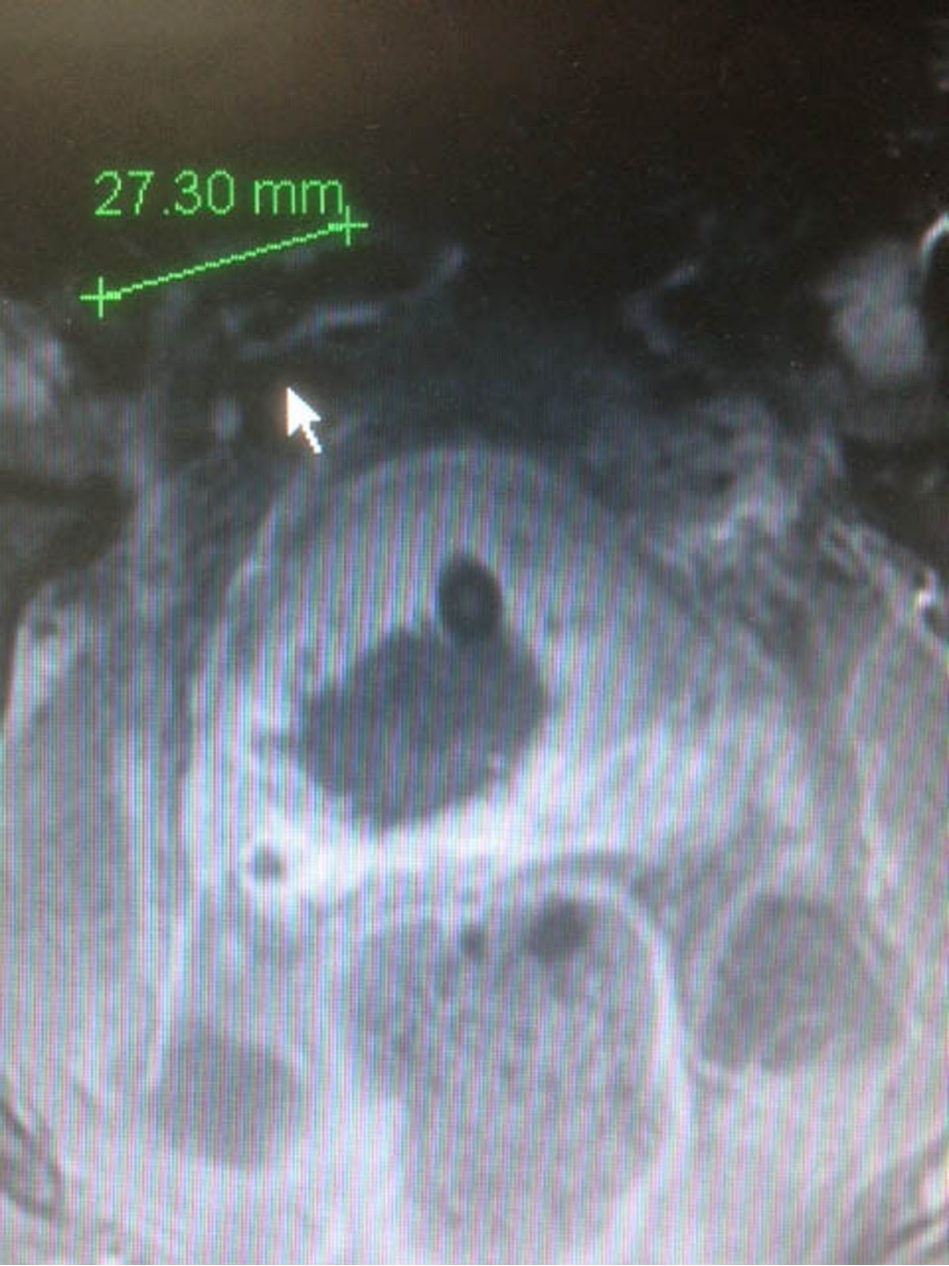
Control MRI after focal HIFU

